# Complete sequences of two novel *bla*_NDM-1_-harbouring plasmids from two *Acinetobacter towneri* isolates in China associated with the acquisition of Tn*125*

**DOI:** 10.1038/s41598-017-09624-0

**Published:** 2017-08-24

**Authors:** Dayang Zou, Yong Huang, Wei Liu, Zhan Yang, Derong Dong, Simo Huang, Xiaoming He, Da Ao, Ningwei Liu, Shengshu Wang, Yong Wang, Yigang Tong, Jing Yuan, Liuyu Huang

**Affiliations:** 1 Institute of Disease Control and Prevention, Academy of Military Medical Sciences, Beijing, China; 2 State Key Laboratory of Pathogen and Biosecurity, Beijing Institute of Microbiology and Epidemiology, Beijing, China

## Abstract

Two novel New Delhi metallo-β-lactamase-1 (NDM-1)-positive plasmids containing a complete composite transposon, Tn*125*, from two respective *Acinetobacter towneri* isolates were characterized. Plasmid pNDM-GJ01 (30,293 bp) isolated from *A*. *towneri* G165 did not show homology to any known plasmid structure, except for the transposon Tn*125* containing *bla*_NDM-1_. A novel *repB* gene and two XRE-type transcriptional regulators were found in pNDM-GJ01. Plasmid pNDM-GJ02 (62,011 bp) isolated from *A*. *towneri* G295 showed the highest homology to pBJAB0715 (41% coverage, 99% nucleotide identity). In addition to the *bla*_NDM-1_-harbouring transposon Tn*125*, pNDM-GJ02 also had an IS*26*-composite transposon, which contains IS*CR1* and two class 1 integrons carrying different cassette arrays. Both clinical isolates were highly resistant to β-lactams and susceptible to tigecycline and colistin. Ten other resistance genes were detected in G295, and one other resistance gene was detected in G165. No transconjugant was obtained from any of the donors by broth and filter mating. The emergence of these two novel plasmids carrying NDM-1 in *Acinetobacter* spp., pNDM-GJ01 and pNDM-GJ02, suggests Tn*125* mobile integration.

## Introduction

The emergence of the *bla*_NDM-1_ gene, which confers gram-negative bacteria with antibiotic resistance, has gained worldwide attention owing to its recent and rapid spread^1–3^. New Delhi metallo-β-lactamase-1 (NDM-1)-positive plasmids have accelerated the process of spread because of their potential for mobility and the fact that they carry multiple resistance genes. Pfeifer *et al*. first reported the genetic context of *bla*_NDM-1_ in a clinical *Acinetobacter baumannii* 161/07 isolate discovered in a German hospital in 2007^4^. The *bla*_NDM-1_ gene was integrated on a new transposon structure (Tn*125*) flanked by two insertion elements of IS*Aba125*. Subsequently, Tn*125* harbouring *bla*_NDM-1_ was found on chromosomes in several multiple-resistant *Acinetobacter* spp. isolates throughout Europe^5, 6^. Furthermore, in 2011, Hu *et al*. reported a *bla*_NDM-1_-bearing plasmid, pNDM-BJ01 (GenBank: JQ001791), isolated from a clinical *A*. *lwoffii* strain in China^7^. This plasmid carried a complete Tn*125* transposon and showed high horizontal transfer ability^7^. Since then, several NDM-1-positive plasmids have been isolated from *Acinetobacter* spp. in China, which showed a similar structure to pNDM-BJ01^8, 9^. These pNDM-BJ01-like plasmids have contributed to the spread of *bla*_NDM-1_ in *Acinetobacter* spp. Here, we report the complete sequences of two novel plasmids, pNDM-GJ01 and pNDM-GJ02, isolated from clinical *Acinetobacter towneri* strains that do not share any similar structure with pNDM-BJ01 except for Tn*125* harbouring the *bla*_NDM-1_ gene. Both plasmids were characterized and their complete sequences were elucidated.

## Results

### Species identification

Strains G165 and G295 were identified as *Kocuria varians* (55%) and *Acinetobacter* sp. (90%) respectively by Vitek® 2 system. Both isolates were identified as *Acinetobacter* sp. by 16S-23S rRNA gene intergenic spacer region sequence comparisons, and identified as *Acinetobacter towneri by* analyzing results of the partial *rpoB* sequence^10, 11^.

### Antimicrobial data

The two clinical strains showed similar susceptibility profiles: they were resistant to broad-spectrum cephalosporins, carbapenems, fluoroquinolones, and sulfamethoxazole. However, they showed a slight difference in susceptibility to aminoglycosides: G165 was resistant to amikacin, and was intermediate to gentamicin and tobramycin; G295 was resistant to amikacin and tobramycin, and was intermediate to gentamicin. Furthermore, both strains were susceptible to tigecycline and colistin, as well as to tetracycline and aztreonam (Table 1)^12^.

**Table 1:** Antimicrobial susceptibility profiles (MICs in mg/L) of NDM-producing *Acinetobacter towneri*.

Isolate	Antibiotics													
	CAZ	CTX	IMP	MEM	AMK	GEN	TOB	CIP	OFX	SMZ	TET	ATM	TIG	COL
G165	>256	>256	16	16	64	8	8	32	32	>256	2	4	0.25	0.5
G295	>256	>256	16	16	128	8	16	8	8	>256	4	4	0.25	0.5

CAZ, ceftazidime; CTX, cefotaxime; IPM, imipenem; MEM, meropenem; AMK, amikacin; GEN, gentamicin; TOB, tobramycin; CIP, ciprofloxacin; OFX, ofloxacin; SMZ, sulfamethoxazole; TET, tetracycline; ATM, aztreonam; TIG, tigecycline; COL, colistin.

Metallo-beta lactamase (MBL) detection with Etest MBL strips (bioMérieux, France) was positive for both strains. Apart from *bla*_NDM-1_, 10 other resistance genes, including *bla*_PSE-1_, *bla*_VIM-11_, *bla*_IMP-1_, *bla*_OXA-58_, *sul1*, *sul2*, *tetA*, *aacC1* gene, *aacC3*, and *aacA4*, were detected in G295, whereas only one other resistance gene, *tetA*, was detected in G165.

### Transconjugation assays

No transconjugants were obtained for the two plasmids, in spite of using three kinds of recipients and applying both methods repeatedly. Subsequent plasmid analysis showed that these two plasmids did not carry the element required to initiate the transconjugation.

### pNDM-GJ01 characteristics

**Figure 1:**
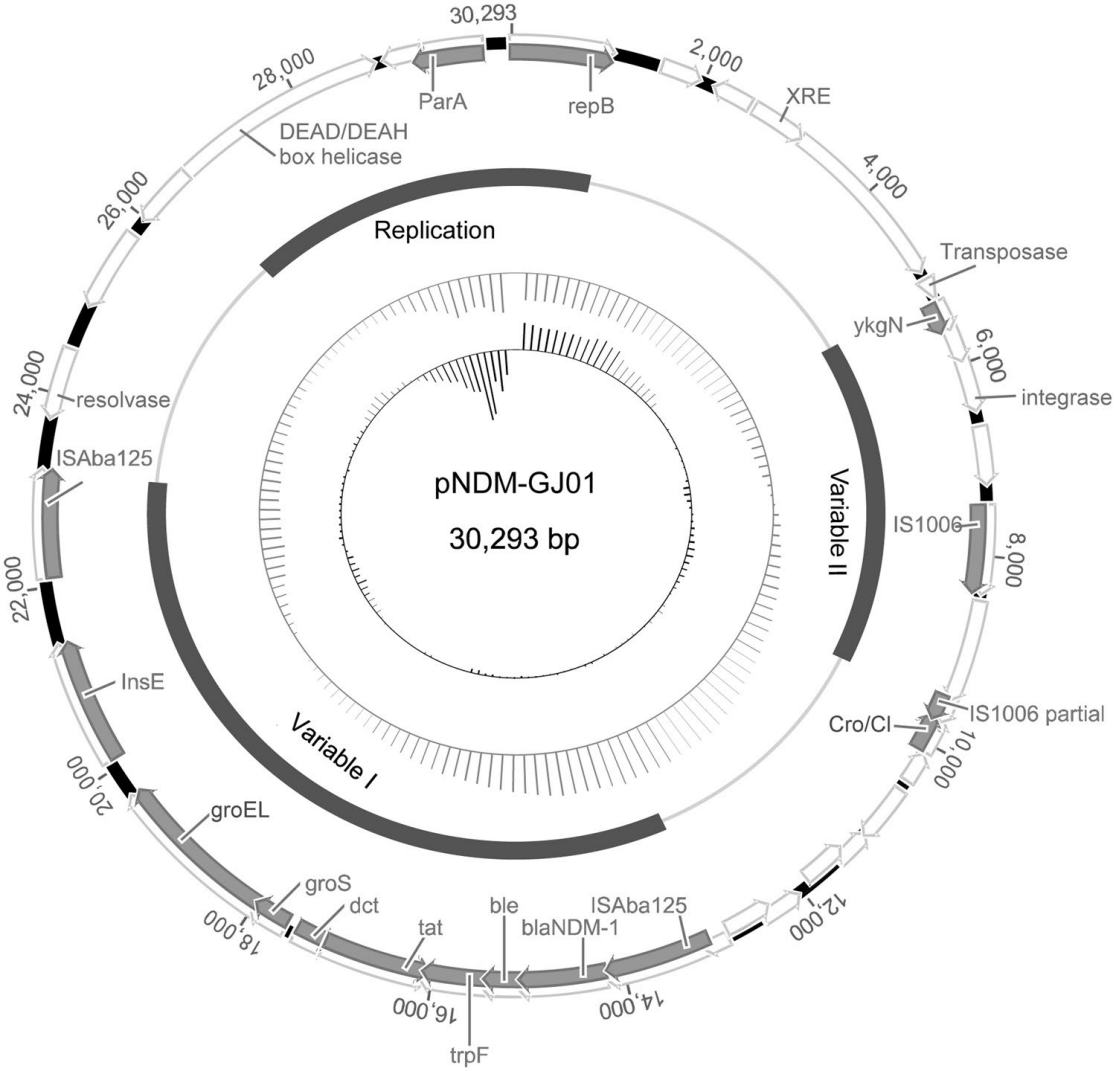
Circular maps of plasmids pNDM-GJ01. Starting from the outside, the first outer circle contains the coordinates of the complete plasmid circle and open reading frames (ORFs). The white arrows represent the direction of transcription. In addition, coding sequences, with known gene names reported in the NCBI database, are indicated by grey arrows. The second circle indicates the functional sequence blocks. The third circle indicates the GC content of plasmids, and the innermost circle indicates the GC-skew of the plasmids.

Plasmid pNDM-GJ01 from the isolate G165 was 30,293 bp long and contained 35 predicted coding sequences with an average GC content of 43.4% (Fig. 1). Three different functional regions were predicted: variable region I containing the complete composite transposon Tn*125*, a replication region, and variable region II containing multiple truncated transposable elements (Fig. 1). Plasmid pNDM-GJ01 was believed to contain a novel structure, as a BLAST search showed that it had no identical structure to previously released plasmids in NCBI, with the exception of Tn*125*. In addition, the plasmid pNDM-GJ01 could not be classified using the PCR-based replicon typing method (PBRT)^13^.

**Figure 2:**
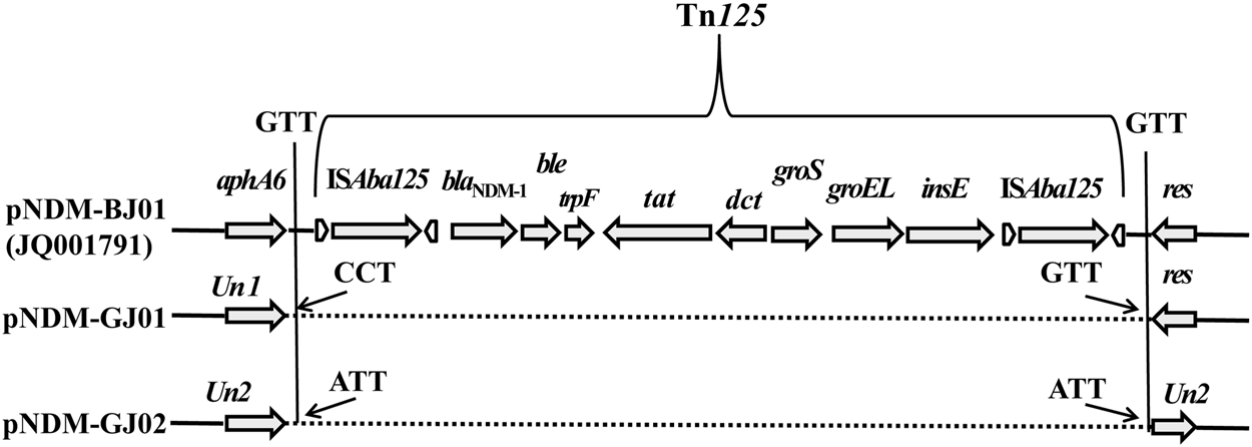
Features of transposon Tn*125* and its target site duplications. Arrows indicate genes and their transcription orientations, and all regions are not drawn to scale. Similar structures and high sequence homology are shown by the dotted line. The 3-bp Tn*125* target sites in each plasmid are indicated by thin arrows. *Un*1 and *Un*2 indicate unknown reading frames encoding hypothetical proteins, and *res* indicates a gene encoding resolvase.

The complete composite transposon Tn*125* was found in variable region I. The Tn*125* sequence was relatively conserved without loss of the downstream segment, showing 99% sequence similarity with that of pNDM-BJ01 (Fig. 1). The gene *aphA6* was not detected in the 5′-flanking region of Tn*125* as in pNDM-BJ01, but the resolvase was retained in the 3′-flanking region^7^. The GC content of Tn*125* was 61% and that of the whole plasmid was 43%, which indicated that the emergence of the NDM-1-harbouring novel plasmid is related to Tn*125* integration and acquisition. Moreover, the target site duplications of Tn*125* were not found in the plasmid; the adjacent base of the left inverted repeat (IRL) sequence of upstream IS*ba125* was CCT and the adjacent base of the right inverted repeat (IRR) sequence of downstream IS*ba125* was GTT (Fig. 2). The replication region contained a DEAD/DEAH box helicase gene, the plasmid-partitioning gene *parA*, and the *repB* gene (Fig. 1). The *repB* gene showed high identity with many sequences encoding an initiator RepB protein from the genome of *Acinetobacter* spp. published in the NCBI database: it showed 99% amino acid identity with a hypothetical protein from *A*. *towneri* strain KCTC 12419 (NCBI Reference Sequence: WP_004968874.1) and 98% amino acid identity with an initiator RepB protein from *Acinetobacter* sp. YZS-X1–1 (NCBI Reference Sequence: WP_034171688.1), which was isolated from the same region as strain G165 (Guangzhou, China). These novel classes of replication proteins are of great significance for plasmid replication and stability^13, 14^. The variable region contained multiple truncated transposable elements and regulatory factors (Fig. 1). An unknown insertion sequence with the downstream fragment missing and IS*1006* may be involved in multiple transposition events. Two regulators (XRE family transcriptional regulator and Cro/Cl family transcriptional regulator) were also found in this region, which is related to the conjugation of plasmids and genomic islands^15^.

### pNDM-GJ02 characteristics

**Figure 3:**
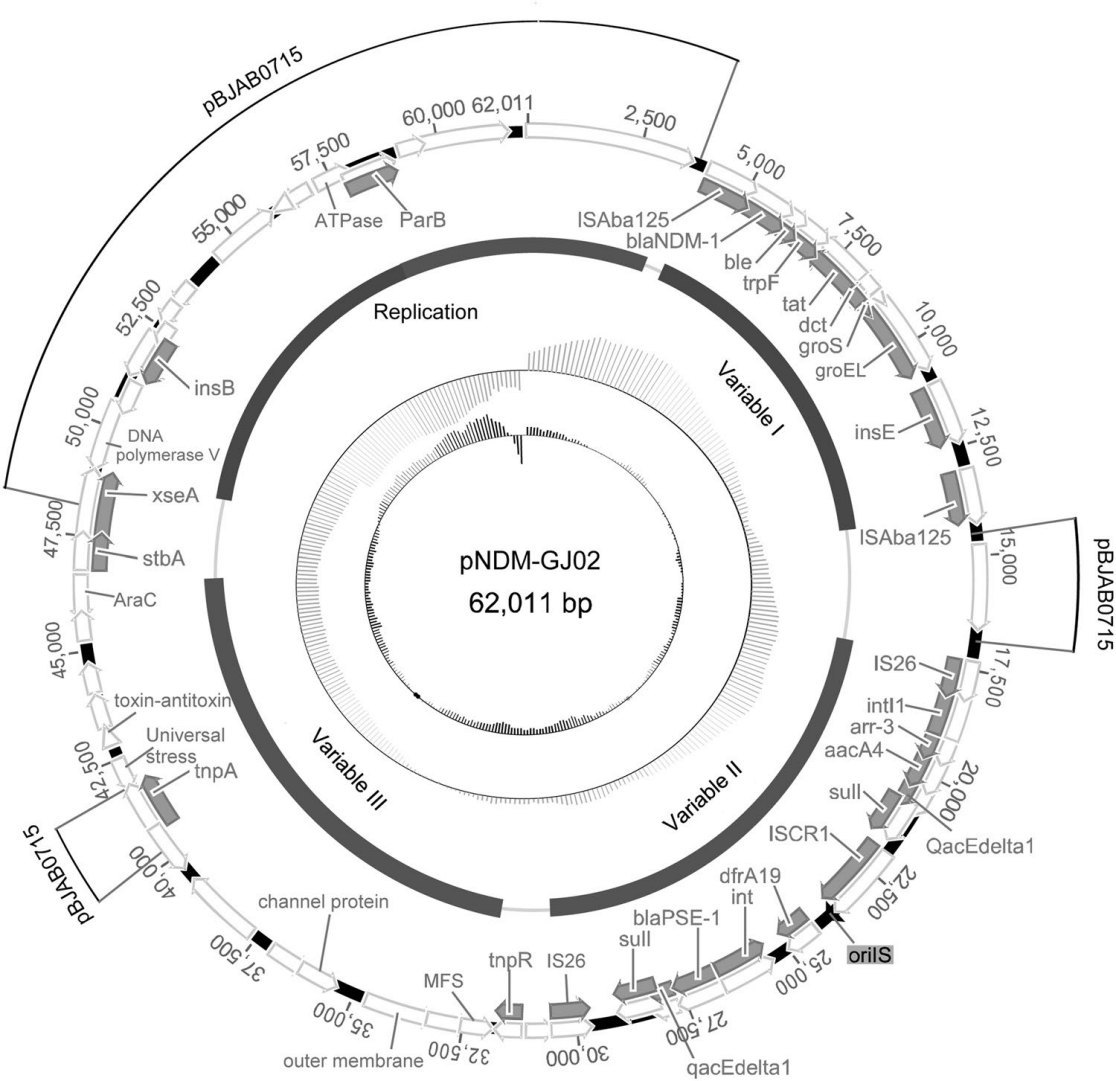
Circular maps of plasmids pNDM-GJ02. The legend of Fig. 3 was same as that of Fig. 1.

Plasmid pNDM-GJ02 from the isolate G295 was 62,011 bp long and contained 58 predicted coding sequences with an average GC content of 45.4% (Fig. 3). Four different functional regions were predicted: variable region I, variable region II, variable region III, and a replication region (Fig. 3). Plasmid pNDM-GJ02 showed the highest similarity to the plasmid pBJAB0715 (41% coverage and 99% nucleotide identity; GenBank: CP003848.1). Based on these results, we concluded that the emergence of pNDM-GJ02 has involved multiple recombination events (Fig. 3). Plasmid pNDM-GJ02 also could not be classified using the PBRT method^13^.

In variable region I, the complete composite transposon Tn*125* was detected. The Tn*125* sequence was also relatively conserved without loss of the downstream segment, showing 99% sequence similarity with that of pNDM-BJ01 (Fig. 3). Tn*125* was inserted into the gene from pBJAB0715 (GenBank: CP003848.1), which encoded a protein of unknown function. The GC content of Tn*125* was 61% and that of the whole plasmid was 45%, which indicated that the emergence of the NDM-1-harbouring novel plasmid was related to Tn*125* integration and acquisition. The 3-bp (ATT) target site duplications of Tn*125* were found in this plasmid (Fig. 2).

In variable region II, the composite transposon flanked by IS*26* was found to be bracketed by two copies of the same insertion sequence with opposite directions (Fig. 3). The class 1 integron was found in both the upstream and downstream regions of the transposon. The gene cassette of the upstream integron was *aar-3-aacA4-qacEdelta1-sul1*, and the integrase gene was truncated by the IS*26* insertion sequence. The downstream integron had a typical structure of 5ʹ- and 3′-conserved regions and contained a *bla*_PSE-1_ gene cassette. A specific type of transposon (IS*CR1*) was found between the two integrons, which has previously been shown to be able to transfer its adjacent sequences and structures (Fig. 3)^16^.

Variable region III comprised multiple transposable elements, regulatory factors, and transport proteins. The resolvase TnpR and the transposase TnpA were detected in the variable region, of which the 5′-flanking region showed a transport structure encoding an MFS family transport permease, channel protein, and outer membrane protein. In addition, genes encoding multiple regulation proteins were identified in this region, including a universal stress protein, antitoxin components belonging to the toxin and antitoxin system, and AraC family transcription factors (Fig. 3).

The replication region contained replication-related functional genes as well as several transposable elements, which may be related to the multiple recombination events of the plasmid. The *rep* gene was not found, and only a gene encoding a novel partitioning protein, parB, was observed in this region. Moreover, we also found genes encoding multiple enzymes associated with plasmid replication and distribution, including an ATP enzyme, DNA polymerase V, and deoxyribonuclease 7 subunits (*xseA*).

## Discussion

Two novel plasmids, pNDM-GJ01 and pNDM-GJ02, containing NDM-1 were found from two *A*. *towneri* isolates in China. The plasmids showed a novel structure and contained a complete composite transposon, Tn*125*. A BLAST search showed no homology with any known plasmid structure in the NCBI database to pNDM-GJ01, except for the transposon Tn*125* containing *bla*_NDM-1_. A region showing high sequence similarity to the plasmid pBJAB0715 (41% coverage, 99% nucleotide identity) and two complete composite transposons (Tn*125*, IS*26*) were found in pNDM-GJ02. It was concluded that the emergence of novel plasmids carrying NDM-1 in *Acinetobacter* spp. is the result of Tn*125* mobile integration involving multiple recombination events.

In *Acinetobacter* spp., transposon Tn*125* appeared to be the main vehicle for dissemination of *bla*_NDM-1_^5^. In *Enterobacteriaceae*, the complete Tn*125* and multiple residual Tn*125*-like structures were found in NDM-1-positive plasmids, they appeared with related mobile factors, such as IS*26*, IS*903*, IS*Kpn14*, IS*1*, IS*Ec33*, and Tn*3*, which participated in the transfer and integration of *bla*_NDM-1_ between different plasmids^17–21^. These results suggest that the *bla*_NDM-1_ gene is spread by transposable elements between different plasmids, and is then further spread in multiple bacteria via plasmids. Over the past two years, an increasing number of reports associated with *Enterobacteriaceae* carrying NDM-1 have begun to emerge continuously in China^22–25^. Given the trend of the spread of the *bla*_NDM-1_ gene in *Enterobacteriaceae*, the potential threat of these dangerous resistant bacteria has not been eliminated. Therefore, further effort should be devoted to detecting the prevalence and spread of *bla*_NDM-1_ in China.

## Materials and Methods

### Ethics statement

All volunteers provided written, informed consent to participate in this study, which was reviewed and approved by the ethics committee of the Academy of Military Medical Sciences, China. All experiments were performed in accordance with relevant guidelines and regulations.

### Bacterial isolates and patient history

*A*. *towneri* G165 and G295 were isolated in 2011 as part of the continuous surveillance on the infection status of NDM-1-producing bacteria in China^26^. Both samples were collected from outpatients. The first case involved a 50-year-old female patient suffering from recurring urinary tract infection. The second case involved a 30-year-old male patient with suspected chronic appendicitis. Both patients received antibiotics and had not travelled to India. Species identification of the isolates was carried out using a Vitek® 2 system (bioMérieux, France), 16S–23S rRNA gene intergenic spacer sequencing, and partial *rpoB* sequence analysis^10, 11^.

### Antimicrobial susceptibility testing

Antimicrobial susceptibility testing was performed by the microbroth dilution method according to the Clinical and Laboratory Standards Institute (CLSI) guidelines. CLSI 2016 breakpoints were applied (M100-S26)^12^.

Detection of MBLs was performed with Etest MBL strips (AB bioMérieux, St. Louis, MO, USA) containing IMP (IP) and IMP + EDTA (IPI). Strips were used according to instructions provided by the manufacturer.

### Molecular detection of β-lactamase production

The putative carbapenemase, ESBL, and AmpC resistance genes were screened by PCR^27–30^. Aminoglycoside resistance genes were also detected by PCR, using the oligonucleotides listed in Supplementary Data Table S1 ^31^. Positive PCR results were confirmed by direct sequencing. Furthermore, all original contigs from the plasmids were searched against the nucleotide database from NCBI to find resistance genes.

### Transconjugation assays

The horizontal transfer capability of the *bla*_NDM-1_ gene was assessed by broth and filter mating using a standard *Escherichia coli* J53 azide-resistant strain, *Shigella flexneri* 2a 301, and *Salmonella* Paratyphi A CMCC 50973 as the recipients^2^. The donor/recipient ratio was 10:1 and the temperature was 30 °C. MacConkey agar containing 100 mg/L sodium azide and 0.5 mg/L meropenem was used to select for *E*. *coli* J53 transconjugants. Both SS agar and XLD media (BD Difco, USA) with 0.5 mg/L meropenem were chosen to select for *S*. *flexneri* and *S*. Paratyphi transconjugants. Putative transconjugants were confirmed by detection of *bla*_NDM-1_ with PCR as described above.

### Plasmid DNA sequencing, annotation, and analysis

Plasmid DNA isolation and purification was performed with the BAC/PAC DNA Kit (Omega Biotek, Norcross, GA, USA) according to the manufacturer’s protocol. Single-end pyrosequencing reads of plasmids were generated using the Ion Torrent™ sequencing platform^32^. Raw reads were first assembled into contigs using Newbler version 2.9, followed by gap filling by local assembly. To ensure accuracy, the raw reads were mapped onto the assembled complete genomes to detect the mis-assembly and low quality regions. Then the genomes were revised and the low quality regions were further confirmed by overlap PCR followed by Sanger sequencing. Each assembled genome was annotated with the Rapid Annotations using Subsystems Technology (RAST) server and verified with the Basic Local Alignment Search Tool (BLAST) against the non-redundant NCBI database (http://blast.ncbi.nlm.nih.gov/Blast.cgi)^33^.

### Nucleotide accession numbers

The complete sequences of pNDM-GJ01 and pNDM-GJ02 have been deposited in GenBank (accession numbers KT965092 and KT965093, respectively).

## Electronic supplementary material

Dataset 1
