# Supplementary material for: Complete sequences of two novel blaNDM-1-harbouring plasmids from two Acinetobacter towneri isolates in China associated with the acquisition of Tn125
Source: Sci Rep. 2017 Aug 24;7:9405. doi: 10.1038/s41598-017-09624-0 (PMC5571222; doi:10.1038/s41598-017-09624-0)
Supplement: Supplementary file 1 — Dataset 1 [file 41598_2017_9624_MOESM1_ESM.docx]

**Complete sequences of two novel *bla*_NDM-1_-harbouring plasmids from two *Acinetobacter towneri* isolates in China associated with the acquisition of Tn*125***

Dayang Zou^a#^, Yong Huang^b#^, Wei Liu^a#^, Zhan Yang, Derong Dong, Simo Huang^a^, Xiaoming He^a^, Da Ao^a^, Ningwei Liu^a^, Shengshu Wang^a^, Yong Wang^a^, Yigang Tong^b^, Jing Yuan^a*^, Liuyu Huang^a*^

^a^Institute of Disease Control and Prevention, Academy of Military Medical Sciences, Beijing, China; ^b^State Key Laboratory of Pathogen and Biosecurity, Beijing Institute of Microbiology and Epidemiology, Beijing, China

*Corresponding author. Institute of Disease Control and Prevention, Academy of Military Medical Sciences, Beijing, China. Tel: +86-10-66948331; Fax: +86-10-66948304; E-mail: huangliuyuly@163.com, yuanjing6216@163.com.

#: These authors contributed equally to this work.

**Running title: New *bla*_NDM-1_-harbouring plasmids in China**

**Keywords: carbapenemases, plasmids, Tn*125*, integration, *Acinetobacter* spp.**

Supplementary data

Table S1. Oligonucleotides used for molecular detection of β-lactamase production and aminoglycoside resistance genes.

| Primer pairs | Sequence | β-Lactamase detected | Product size (bp) | References |
| --- | --- | --- | --- | --- |
| 1 | 5'-GGAATAGAGTGGCTTAAyTCTC-3'  5'-CCAAACyACTACGTTATCTkGAG-3' | IMP | 188 | ^1^ |
| 2 | 5'-GATGGTGTTTGGTCGCATA-3'  5'-CGAATGCGCAGCACCAG-3' | VIM | 390 | ^1^ |
| 3 | 5'-CTTGCTGCCGCTGTGCTG-3'  5'-GCAGGTTCCGGTTTTGTCTC-3' | KPC | 489 | ^2^ |
| 4 | 5'-GATGTGTCATAGTATTCGTCGT-3'  5'-TCACAACAACTAAAAGCACTGT-3' | OXA-23-like | 1058 | ^3^ |
| 5 | 5'-ATGAAAAAATTTATACTTCCTATATTCAGC-3'  5'-TTAAATGATTCCAAG ATTTTCTAGC-3' | OXA-24-like | 828 | ^3^ |
| 6 | 5'-TAATGCTTTGATCGGCCTTG-3'  5'-TGGATTGCACTTCATCTTGG-3' | OXA-51-like | 353 | ^4^ |
| 7 | 5'-AAGTATTGGGGCTTGTGCTG-3'  5'-CCCCTCTGCGCTCTACATAC-3' | OXA-58-like | 599 | ^4^ |
| 8 | 5'-TACAAGGGATTCGGCATCG-3'  5'-TAATGGCCTGTTCCCATGTG-3' | SIM | 570 | ^5^ |
| 9 | 5'-CCTACAATCTAACGGCGACC-3'  5'-TCGCCGTGTCCAGGTATAAC-3' | SPM | 648 | ^6^ |
| 10 | 5'-CTTGTAGCGTTGCCAGCTTTA-3'  5'-CAGCCCAAGAGCTAATTGAGG-3' | GIM | 562 | ^7^ |
| 11 | 5'-TGGTTATGCGTTATATTCGCC-3'  5'-GGTTAGCGTTGCCAGTGCT-3' | SHV | 868 | ^8^ |
| 12 | 5'-TCCGCTCATGAGACAATAACC-3'  5'-TTGGTCTGACAGTTACCAATGC-3' | TEM | 931 | ^9^ |
| 13 | 5'-TCTTCCAGAATAAGGAATCCC-3'  5'-CCGTTTCCGCTATTACAAAC-3' | CTX-M | 909 | ^9^ |
| 14 | 5'-AAGAAACGCTACTCGCCTGC-3'  5'-CCACTCAACCCATCCTACCC-3' | OXA-2 group | 478 | ^10^ |
| 15 | 5'-GTCTTTCGAGTACGGCATTA-3'  5'-ATTTTCTTAGCGGCAACTTAC-3' | OXA-10 group | 720 | ^11^ |
| 16 | 5'-ATGAATGTCATCACAAAATG-3'  5'-TCAATCCGGACTCACT-3' | PER | 927 | ^12^ |
| 17 | 5'-ATGCGCTTCATTCACGCAC-3'  5'-CTATTTGTCCGTGCTCAGG-3' | GES | 864 | ^13^ |
| 18 | 5'-CGACTTCCATTTCCCGATGC-3'  5'-GGACTCTGCAACAAATACGC-3' | VEB | 644 | ^13^ |
| 19 | 5'-ACCGTATTGAGCCTGATTTA-3'  5'- ATTGAAGCCTGTGTTTGAGC-3′ | PSE group | 321 | ^14^ |
| 20 | 5'-TTTCTCCTGAACGTGGCTGGC-3'  5'-TGGCCAGAACTGACAGGCAAA-3' | CMY-2 | 462 | ^15^ |
| 21 | 5'-AACTTTCACAGGTGTGCTGGGT-3'  5'-CCGTACGCATACTGGCTTTGC-3' | DHA-1 | 405 | ^15^ |
| 22 | 5'-CCAAGAGCAATAAGGGCATA-3'  5'-CACTATCATAACCACTACCG-3' | aac(6’)/aph(2”) | 220 | ^16^ |
| 23 | 5'- ATGACTGAGCATGACCTTGC-3'  5'-TTAGGCATCACTGCGTGTTC-3' | aac(6')-Ib | 519 | ^17^ |
| 24 | 5'-ACCTACTCCCAACATCAGCC-3'  5'-ATATAGATCTCACTACGCGC-3' | aac(3)-I | 169 | ^18^ |
| 25 | 5'- GGGGTCTTACTATTCTGCCT-3'  5'-ATTCCCTTCTCCTTTCCAG-3' | armA | 504 | This study |

**References**

1 Ellington, M. J., Kistler, J. J., Livermore, D. M. & Woodford, N. Multiplex PCR for rapid detection of genes encoding acquired metallo-β-lactamases. *Journal of Antimicrobial Chemotherapy* **59**, 321-322 (2007).

2 Tenover, F. C. *et al.* Carbapenem resistance in Klebsiella pneumoniae not detected by automated susceptibility testing. *Emerging infectious diseases* **12**, 1209-1213 (2006).

3 Jeon, B. *et al.* Investigation of a Nosocomial Outbreak of Imipenem-Resistant Acinetobacter baumannii Producing the OXA-23 β-Lactamase in Korea. *Journal of clinical microbiology* **43**, 2241-2245 (2005).

4 Turton, J. F. *et al.* Identification of Acinetobacter baumannii by Detection of the blaOXA-51-like Carbapenemase Gene Intrinsic to This Species. *Journal of clinical microbiology* **44**, 2974-2976 (2006).

5 Zhou, H. *et al.* Dissemination of imipenem-resistant Acinetobacter baumannii strains carrying the ISAba1–blaOXA-23 genes in a Chinese hospital. *Journal of medical microbiology* **56**, 1076-1080 (2007).

6 Gales, A. C., Menezes, L. C., Silbert, S. & Sader, H. S. Dissemination in distinct Brazilian regions of an epidemic carbapenem-resistant Pseudomonas aeruginosa producing SPM metallo-β-lactamase. *Journal of Antimicrobial Chemotherapy* **52**, 699-702 (2003).

7 Jin, H., Xu, X.-m., Mi, Z.-h., Mou, Y. & Liu, P. Drug-resistant gene based genotyping for Acinetobacter bauinannii in tracing epidemiological events and for clinical treatment within nosocomial settings. *Chinese Medical Journal (English Edition)* **122**, 301 (2009).

8 Kim, J., Kwon, Y., Pai, H., Kim, J. & Cho, D. Survey of Klebsiella pneumoniae Strains Producing Extended-Spectrum β-Lactamases: Prevalence of SHV-12 and SHV-2a in Korea. *Journal of clinical microbiology* **36**, 1446-1449 (1998).

9 Sturenburg, E., Lang, M., Horstkotte, M. A., Laufs, R. & Mack, D. Evaluation of the MicroScan ESBL plus confirmation panel for detection of extended-spectrum beta-lactamases in clinical isolates of oxyimino-cephalosporin-resistant Gram-negative bacteria. *The Journal of antimicrobial chemotherapy* **54**, 870-875, doi:10.1093/jac/dkh449 (2004).

10 Bert, F., Branger, C. & Lambert-Zechovsky, N. Identification of PSE and OXA beta-lactamase genes in Pseudomonas aeruginosa using PCR-restriction fragment length polymorphism. *The Journal of antimicrobial chemotherapy* **50**, 11-18 (2002).

11 Vahaboglu, H. *et al.* Practical approach for detection and identification of OXA-10-derived ceftazidime-hydrolyzing extended-spectrum β-lactamases. *Journal of clinical microbiology* **36**, 827-829 (1998).

12 Celenza, G. *et al.* Spread of bla(CTX-M-type) and bla(PER-2) beta-lactamase genes in clinical isolates from Bolivian hospitals. *The Journal of antimicrobial chemotherapy* **57**, 975-978, doi:10.1093/jac/dkl055 (2006).

13 Poirel, L., Le Thomas, I., Naas, T., Karim, A. & Nordmann, P. Biochemical sequence analyses of GES-1, a novel class A extended-spectrum beta-lactamase, and the class 1 integron In52 from Klebsiella pneumoniae. *Antimicrobial agents and chemotherapy* **44**, 622-632 (2000).

14 Wroblewska, M. M. *et al.* Outbreak of nosocomial meningitis caused by Acinetobacter baumannii in neurosurgical patients. *Journal of Hospital Infection* **57**, 300-307 (2004).

15 Perezperez, F. J. & Hanson, N. D. Detection of Plasmid-Mediated AmpC β-Lactamase Genes in Clinical Isolates by Using Multiplex PCR. *Journal of clinical microbiology* **40**, 2153-2162 (2002).

16 Van Asselt, G. J., Vliegenthart, J. S., Petit, P. L. C., De Klundert, J. A. V. & Mouton, R. P. High-level aminoglycoside resistance among enterococci and group A streptococci. *Journal of Antimicrobial Chemotherapy* **30**, 651-659 (1992).

17 Jones, G. L. *et al.* Prevalence and distribution of plasmid-mediated quinolone resistance genes in clinical isolates of Escherichia coli lacking extended-spectrum β-lactamases. *Journal of Antimicrobial Chemotherapy* **62**, 1245-1251 (2008).

18 Van de Klundert, J. PCR detection of genes coding for aminoglycoside-modifying enzymes. *Diagnostic molecular microbiology: principles and applications* (1993).
